# Serotonin 1A Receptor Binding of [^11^C]CUMI-101 in Bipolar Depression Quantified Using Positron Emission Tomography: Relationship to Psychopathology and Antidepressant Response

**DOI:** 10.1093/ijnp/pyac001

**Published:** 2022-01-06

**Authors:** Martin J Lan, Francesca Zanderigo, Spiro P Pantazatos, M Elizabeth Sublette, Jeffrey Miller, R Todd Ogden, J John Mann

**Affiliations:** Department of Psychiatry, Vagelos College of Physicians and Surgeons at Columbia University, New York, NY, USA; Molecular Imaging and Neuropathology Area, New York State Psychiatric Institute, New York, NY, USA; Department of Psychiatry, Vagelos College of Physicians and Surgeons at Columbia University, New York, NY, USA; Molecular Imaging and Neuropathology Area, New York State Psychiatric Institute, New York, NY, USA; Department of Psychiatry, Vagelos College of Physicians and Surgeons at Columbia University, New York, NY, USA; Molecular Imaging and Neuropathology Area, New York State Psychiatric Institute, New York, NY, USA; Department of Psychiatry, Vagelos College of Physicians and Surgeons at Columbia University, New York, NY, USA; Molecular Imaging and Neuropathology Area, New York State Psychiatric Institute, New York, NY, USA; Department of Psychiatry, Vagelos College of Physicians and Surgeons at Columbia University, New York, NY, USA; Molecular Imaging and Neuropathology Area, New York State Psychiatric Institute, New York, NY, USA; Department of Psychiatry, Vagelos College of Physicians and Surgeons at Columbia University, New York, NY, USA; Molecular Imaging and Neuropathology Area, New York State Psychiatric Institute, New York, NY, USA; Department of Psychiatry, Vagelos College of Physicians and Surgeons at Columbia University, New York, NY, USA; Molecular Imaging and Neuropathology Area, New York State Psychiatric Institute, New York, NY, USA; Department of Radiology, Vagelos College of Physicians and Surgeons at Columbia University, New York, NY, USA

**Keywords:** Antidepressant, bipolar disorder, positron emission tomography, selective serotonin reuptake inhibitor, serotonin

## Abstract

**Background:**

The pathophysiology of bipolar disorder (BD) remains largely unknown despite it causing significant disability and suicide risk. Serotonin signaling may play a role in the pathophysiology, but direct evidence for this is lacking. Treatment of the depressed phase of the disorder is limited. Previous studies have indicated that positron emission tomography (PET) imaging of the serotonin 1A receptor (5HT1AR) may predict antidepressant response.

**Methods:**

A total of 20 participants with BD in a current major depressive episode and 16 healthy volunteers had PET imaging with [^11^C]CUMI-101, employing a metabolite-corrected input function for quantification of binding potential to the 5HT1AR. Bipolar participants then received an open-labeled, 6-week clinical trial with a selective serotonin reuptake inhibitor (SSRI) in addition to their mood stabilizer. Clinical ratings were obtained at baseline and during SSRI treatment.

**Results:**

Pretreatment binding potential (BP_F_) of [^11^C]CUMI-101 was associated with a number of pretreatment clinical variables within BD participants. Within the raphe nucleus, it was inversely associated with the baseline Montgomery Åsberg Rating Scale (*P* = .026), the Beck Depression Inventory score (*P* = .0023), and the Buss Durkee Hostility Index (*P* = .0058), a measure of lifetime aggression. A secondary analysis found [^11^C]CUMI-101 BP_F_ was higher in bipolar participants compared with healthy volunteers (*P* = .00275). [^11^C]CUMI-101 BP_F_ did not differ between SSRI responders and non-responders (*P* = .907) to treatment and did not predict antidepressant response (*P* = .580). Voxel-wise analyses confirmed the results obtained in regions of interest analyses.

**Conclusions:**

A disturbance of serotonin system function is associated with both the diagnosis of BD and its severity of depression. Pretreatment 5HT1AR binding did not predict SSRI antidepressant outcome.

The study was listed on clinicaltrials.gov with identifier NCT02473250.

**Significance Statement**

Bipolar disorder is a cause of substantial disability and suicide risk. The pathophysiology of the disorder is not known, limiting novel treatment development. Serotonin signaling is implicated in the pathophysiology, and medications that alter monoamine signaling can treat both the depressed and manic phases. Serotonin firing and release are regulated by the serotonin 1A receptor (5HT1AR), and brain imaging with positron emission tomography (PET) can measure 5HT1AR levels. Previous PET studies indicated that the 5HT1AR levels may predict antidepressant response. Here, we found that the levels of the 5HT1AR were inversely associated with both the severity of depression and the level of lifetime aggression in bipolar depression. 5HT1AR levels also differed between bipolar participants and healthy volunteers. These data provide direct evidence that serotonin signaling is important to the pathophysiology of bipolar disorder. We found no association between baseline 5HT1AR PET signal with treatment outcome to an antidepressant, however.

## Introduction

The pathophysiology of bipolar disorder (BD) remains largely unknown (McIntyre et al., 2020). It is a major cause of disability and carries a high risk for suicide (Merikangas et al., 2011; Pompili et al., 2013). The depressed phase of BD is associated with the most disability and suicide risk (Baldessarini et al., 2010). Treatment of the depressed phase is limited to 4 Food and Drug Administration–approved medications (quetiapine, lurasidone, cariprazine, and the olanzapine-fluoxetine combination pill) that are not universally effective and can have problematic side effects (Kadakia et al., 2021). Treatment is a trial-and-error process, as there are no predictors of medication response. Optimizing treatment therefore can be a drawn-out process. Understanding the pathophysiology of the disorder could help to advance medication development. A biomarker of response to a specific medication class would help to choose the medication type for the individual patient.

A role for serotonin signaling in the pathophysiology of BD has been proposed (Soares, 2016). Pharmacological manipulation of monoamine signaling is used to treat both the depressive and manic phases, consistent with the importance of monoamine signaling in BD’s pathophysiology (Tohen et al., 2003; Heijnen et al., 2015; Kadakia et al., 2021; Kishi et al., 2021). The serotonin 1A receptor (5HT1AR) is central to serotonin signaling. In the raphe nucleus (RN), where brain serotonin neurons originate, the 5HT1AR is an inhibitory somatodendritic autoreceptor, regulating serotonin neuron firing rate and release (Barnes and Sharp, 1999; Adell et al., 2002). In other brain regions, the 5HT1AR is a postsynaptic serotonin receptor, mediating signaling of target neurons by serotonin (Kia et al., 1996; Varnas et al., 2004). The levels and activity of 5HT1ARs in the brain are regulated in response to changes in brain serotonin signaling, including treatment with selective serotonin reuptake inhibitors (SSRIs; Aznavour et al., 2006; Gray et al., 2013; Kulikov et al., 2018). Positron emission tomography (PET) with 5HT1AR selective radiotracers can be used to quantify binding to the receptor in the brain (Mathis et al., 1994; Milak et al., 2010).

Previous studies provided evidence that 5HT1AR PET imaging may reflect symptoms of BD. PET studies in healthy volunteers (HVs) indicated that 5HT1AR binding was inversely associated with both lifetime aggression and anxiety symptoms (Tauscher et al., 2001; Parsey et al., 2002; Rabiner et al., 2002; Witte et al., 2009). These symptoms were chosen a priori for analysis in part because they comprise the Research Domain Criteria from the National Institute of Mental Health related to serotonin signaling, “frustrative non-reward” and “acute threat.” We previously reported higher binding potential (BP_F_) of [^11^C]WAY-100 635 in the brain of depressed BD participants when compared with HVs (Sullivan et al., 2009), indicating it may be involved with the genesis of depressive symptoms.

SSRIs are frequently used on an off-label basis to treat depression in BD, although their efficacy in BD has not been universally replicated (Goldberg and Nassir Ghaemi, 2005; Baldessarini et al., 2007; Sidor and MacQueen, 2012). One SSRI, fluoxetine, is part of an approved medication for depression in BD when it is combined with olanzapine (Cohn et al., 1989; Tohen et al., 2003). We previously reported that BD participants who had remitted from their depression after 3 months of naturalistic treatment had higher pretreatment brain binding potential of [^11^C]WAY-100 635 compared with those who had not remitted (Lan et al., 2013). The participants in that study had received a variety of medications, including SSRIs. Other studies have also found prediction of antidepressant response by 5HT1AR PET imaging (Meltzer et al., 2004; Parsey et al., 2006a; Moses-Kolko et al., 2007; Miller et al., 2013; Ananth et al., 2020), although one showed no prediction of response to electroconvulsive therapy (Lanzenberger et al., 2013).

Based on these previous studies, we conducted a clinical trial of a SSRI when added to a mood stabilizer in participants with depression and a diagnosis of BD. Participants had PET imaging with the 5HT1AR radiotracer, [^11^C]CUMI-101, before treatment and had depression severity, lifetime aggression, and anxiety severity measured at that time. We hypothesized that 5HT1AR binding potential, quantified by PET imaging with [^11^C]CUMI-101, would have an inverse correlation with aggression and anxiety in bipolar depression. We also hypothesized that higher binding of [^11^C]CUMI-101 would predict treatment response to the SSRI treatment. In addition, we performed a secondary analysis to compare [^11^C]CUMI-101 binding in BD participants with HVs, hypothesizing that we would replicate our finding of higher 5HT1AR PET binding in BD.

## METHODS

### Participants

Forty BD participants were evaluated for the study; 18 were ruled out because of clinical response to divalproex (n = 2), intolerance to the divalproex optimization (n = 3), lack of follow-up after consent (n = 9), or failure to meet inclusion criteria on further evaluation (n = 4). Twenty-two participants who met DSM-IV criteria for BD I or II disorder and were in a major depressive episode received neuroimaging, and 20 participants completed the study and had data analyzed. Diagnosis involved psychiatric interview and Structured Clinical Interview for Axis I disorders by MA- or PhD-level psychologists. Diagnoses were confirmed through a consensus discussion of clinicians that included a research psychiatrist. Participants were aged 18–65 years and had a 17-item Hamilton Depression Rating Scale score >15, with atypical symptoms items included, at the time of enrollment. Participants were excluded from the study if they had an unstable medical condition, history of either substance abuse within 3 months of evaluation or substance dependence within 6 months, positive urine toxicology test, positive pregnancy test, planned pregnancy, or use of 3,4-methylendioxymethamphetamine more than 3 times. They could not have had previous failed trials of 3 serotonin-based antidepressant medications (either an SSRI or serotonin and norepinephrine reuptake inhibitor) as per patient report. They could not have had previous intolerance to any study medication. The study was listed on clinicaltrials.gov with identifier NCT02473250. A total 16 HVs were recruited as part of other studies with different scientific aims (Melhem et al., 2021; Schneck et al., 2021). They had the same inclusion and exclusion criteria, but they had no axis I diagnoses and no first-degree family history of mood disorders, psychotic disorders, or suicide. Written informed consent was obtained for all participants after a description of their study, and all PET scans were performed between July 2014 and October 2019. The study was approved by the Institutional Review Board of the New York State Psychiatric Institute in accordance with the Helsinki Declaration of 1975.

### Clinical Treatment

If BD participants were taking either an anticonvulsant mood stabilizer or lithium at enrollment, they remained on that medication for the study at doses that were within a therapeutic range. If they were not on a mood stabilizer medication, they started divalproex, and the dose was titrated to a therapeutic serum level (40–120 mg/mL). The participants who started divalproex received a 3-week treatment with the medication before PET scanning. If those participants had a 17-item Hamilton Depression Rating Scale score <16, including atypical depression items, 1 week before their scheduled PET scan, they did not proceed with research procedures. If any BD participants were taking psychiatric medications other than the approved mood stabilizers on enrollment, these were tapered off, and those participants had a 3-week period without them before PET scanning. Participants were allowed to take short acting benzodiazepines and hypnotics.

After PET scanning, BD participants received SSRI treatment for 6 weeks in addition to their mood stabilizer. Participants were offered fluoxetine first but could opt to take citalopram if fluoxetine was not clinically warranted. Nineteen BD participants took fluoxetine and 1 took citalopram. Dosing for either of these SSRIs was as follows: the SSRI was started at 20 mg/d; at week 2, if BD participants had not had a full clinical response to the SSRI and did not have dose-limiting side effects, the SSRI dose was increased to 40 mg/d. The dose could be adjusted at any point in the clinical trial between 10 and 40 mg/d as per clinical considerations. Participants met with their psychiatrist weekly, at which time pill counts were performed.

The Montgomery Åsberg Depression Rating Scale (MADRS) was the primary clinical outcome measure and was measured at weeks 0, 2, 4, and 6 in BD participants. Participants completed the Beck Depression Inventory (BDI), Hamilton Anxiety Rating Scale (HAM-A), and Young Mania Rating Scale within 1 week of PET imaging and the Buss Durkee Hostility Inventory (BDHI) during the study.

To calculate the trajectory of depression improvement, a curve was fitted for each participant’s MADRS scores over the 6-week treatment course using a previously published method that provides information on the slope and curvature of the treatment response (Tarpey et al., 2003). This approach incorporates depression severity data from all the time points in the clinical trial instead of considering just the initial and final depression severity points. The extrapolated values from those curves at the baseline timepoint and the last MADRS measurement were used to quantify response (Lan et al., 2017).

### Radiochemistry and Neuroimaging

[^11^C]CUMI-101, [O-methyl -^11^C]2-(4-(4-(2-methoxyphenyl)piperazin-1-yl)butyl)-4-methyl-1,2,4-triazine-3,5(2H,4H)dione, was synthesized as previously described (Milak et al., 2010; Majo et al., 2013). [^11^C]CUMI-101 was injected at <6 mCi per participant in a single bolus. Dynamic PET images were acquired in 3D list mode over 120 minutes using 21 frames of increasing duration (3 × 20 seconds, 3 × 1 minute, 3 × 2 minutes, 2 × 5 minutes, and 10 × 10 minutes) on a Siemens Biograph PET/CT (Siemens, Knoxville, TN, USA). A venous blood sample was obtained from an i.v. line in the contralateral arm to tracer injection to measure the tracer’s total radioactivity in plasma, unmetabolized parent fraction, and plasma free fraction (f_P_) after decay correction (Parsey et al., 2000). The sample was drawn at 60 minutes after injection, although 8 HV participants had it drawn at 40 minutes. These time points for anchoring the simultaneous estimation of input function have been found to lead to similar estimates of [^11^C]CUMI-101 binding in a study where within-patient venous and arterial blood data were collected (Bartlett et al., 2019). A T1-weighted structural fast spoiled gradient echo magnetic resonance image (MRI) was obtained on a GE SIGNA Premier 3T scanner with a 32-channel head coil (General Electric, Fairfield, CT, USA) for co-registration to the PET data. Mean injected mass was 2.00 μg (±1.02) and mean injected dose was 11.81 mCi (±3.41).

### PET Image Processing

Attenuation correction was performed with a low-dose computer tomography scan. Reconstruction used a filtered back projection to a 256 × 256 matrix (voxel size: 1 mm × 1 mm × 2 mm), using a Shepp 0.5 filter (2 mm in full width at half maximum) and zoom factor of 3.2. Functional magnetic resonance imaging of the brain linear image registration tool (FLIRT), version 5.0 (FMRIB Image Analysis Group, Oxford, UK), was used to correct for participant motion by co-registering each PET frame to the eighth frame. The summed PET image frames were co-registered to each participant’s T1-weighted MRI using FLIRT (Delorenzo, 2009). The transformation was then applied to all individual PET frames. Images were resliced in FLIRT with trilinear interpolation and the results verified by visual inspection. Thirteen regions of interest (ROIs) with high levels of 5HT1AR binding were delineated (Sullivan et al., 2009) using in-house software built in MATLAB and an automated algorithm that assigns probabilistic ROIs to a T1-weighted MRI (Parsey et al., 2006b; Milak et al., 2010). Because the RN cannot be reliably identified on structural MRI images, this ROI was labeled using a mask of the average RN location in 52 HVs as determined from [^11^C]WAY100635 voxel binding maps warped into standard space (Delorenzo et al., 2013). ROIs were grey matter-masked. The cerebellar grey matter (CER) was used as the reference region. No partial volume correction was applied. Regional total distribution volume (V_T_) values of [^11^C]CUMI-101 were calculated using Likelihood Estimation in Graphical Analysis (Ogden, 2003) and a simultaneously estimated input function with the single venous blood sample as the anchor, as previously validated (Ogden et al., 2010; Bartlett et al., 2019). The anchor was obtained by multiplying the tracer’s total radioactivity level in plasma by the corresponding parent fraction. Likelihood Estimation in Graphical Analysis was applied to the time activity curves (TACs) using, at each data point in time, a weight equal to the square root of the corresponding acquisition frame duration and a t* = 45 minutes post-injection (Milak et al., 2010). Time activity curves (TACs) were not corrected for vascular contribution, as multiple measures of radiotracer total radioactivity in whole blood were not available. The BP_F_ was then calculated in each ROI as (V_T,ROI_ − V_T,CER_)/f_P_, with V_T,CER_ the volume of distribution estimated in the reference region. BP_F_ is an estimate of B_avail_/K_D_, where B_avail_ is the number of receptors available to bind to the radiotracer and 1/K_D_ is the affinity of the radiotracer for the receptor. MATLAB R2016b and in-house software (*BrainFit*) were used for quantification of PET outcome measures. BP_F_ maps were then obtained by quantifying V_T_ in each voxel using Empirical Bayesian Estimation in Graphical analysis and the same simultaneously estimated input function used at the ROI level (Zanderigo et al., 2010). Corresponding BP_F_ maps were then obtained by calculating in each voxel BP_F_ = (VT − VT_,ref_)/f_P_, with V_T_ the tracer total distribution volume estimated by Empirical Bayesian Estimation in Graphical analysis in each voxel and V_T,ref_ the average V_T_ values of the voxels within the reference region. Each participant’s BP_F_ map was then co-registered to the Montreal Neurological Institute space.

### Statistics

Binding potential measures were analyzed first for the RN by fitting a linear model with log(BP_F_) as outcome. Predictors included relevant pretreatment clinical variables. Age and sex were considered as covariates, and they were removed from the final model if they were not significant. For analysis of data from multiple regions, a similar modeling strategy was taken but fitting linear mixed effect models with participant as the random effect, and region was also included as a fixed effect. Prediction of clinical response was modeled linearly with the end extrapolated MADRS score as outcome, covarying for initial extrapolated MADRS score and imaging or clinical predictors. Demographic or clinical differences between clinical groups were calculated with Student’s *t* test or Fisher’s exact test. Associations between outcome measure components and clinical variables were calculated using Pearson correlation. Significance was defined as *P* < .05, and all tests were 2 sided. SPSS 12 for Mac OSX (www.spss.com) or R (www.R-project.org) was used for calculations.

### Whole**-**Brain Voxel-Wise Analyses

Voxel-level analyses were conducted to validate ROI findings and to delineate the regional distribution of the significant associations. Voxel-level PET BP_F_ maps were spatially normalized and interpolated to 2 × 2 × 2 mm voxel resolution, smoothed with an 8-mm Gaussian kernel, and submitted to separate second-level multiple regression models with the following regressors of interest: (1) differences between BD and HV participants; (2) association with BDI score within BD participants; (3) association with BDHI scores within BD participants; (4) association with HAMA scores within BD participants; and (5) BD participant responders to SSRI compared with non-responders. Sex and overall mean were included as nuisance regressors, and all regressors were mean-centered. An absolute threshold was applied to remove voxels with BP_F_ values <5, and non-gray matter voxels were excluded from analyses via a gray matter mask generated by thresholding a tissue probability map in Montreal Neurological Institute space (provided with SPM8) at >0.2. The cerebellum, as defined using the WFU Pick atlas, was excluded from the analysis. Analyses were conducted using SPM12 (www.fil.ion.ucl.ac.uk/spm/) and implemented in MATLAB version 7.13 on Ubuntu Linux OS 14.0.4. Threshold-free cluster extent correction was applied using permutation inference as implemented in the TFCE toolbox v1.0 r222 from June 30, 2021, for the contrasts of interest and their inverse in each model (all default settings and *P* = 5000 permutations). Non-parametric threshold-free cluster extent statistical maps were generated and threshold at *P* < .05 family wise error corrected, k > 10.

## RESULTS

### Participant Demographics

Demographic variables can be found in Table 1. Baseline BDI scores were obtained for 19/20 BD participants, BDHI for 15/20, and HAM-A was obtained for 18/20. There were 15/20 BD participants taking divalproex at the time of PET scan; 2/20 were taking lithium, 2/20 were taking lamotrigine, and 1/20 were taking oxcarbazepine. One participant lost contact after their PET scan, and 1 was found to be not reliable and was dropped from consideration.

**Table 1.** Clinical Variables for the Participants

	BD Total(n = 20)	BD Responders (n = 7)	BD Nonresponders (n = 13)	*P* value
Age	40.5 (±13.7)	37.1 (±15.9)	42.3 (±12.7)	.44^a^
Sex (% Female)	12 (60%)	4(57%)	8(62%)	1^b^
BDI	25.7 (±12.6)	24.7 (±16.7)	26.3(±10.3)	.80^a^
MADRS initial	27 (± 8.8)	28.7 (±10.8)	26.1(±7.9)	.54^a^
BDHI	35.1 (±10.2)	30.2(±10.0)	37.6(±9.8)	.19^a^
HAMA	15.8 (±6.9)	17.6(±4.8)	15.2(±7.6)	.46^a^
YMRS	3.4 (±2.4)	3.6 (±2.2)	3.3 (±2.6)	.82^a^
Mood stabilizer (% on VPA)	15/20 (75%)	5/7 (71%)	10/13 (77%)	1^b^
Alcohol	7/20 (35%)	3/7 (42.9%)	5/13 (38.4%)	1^b^
Cocaine	1/20 (5%)	0/7 (0%)	1/13 (7.7%)	1^b^
Cannabis	2/20 (10%)	1/7 (14.3%)	1/13 (7.7%)	1^b^
Polysubstance	1/20 (5%)	0/7 (0%)	1/13 (7.7%)	1^b^
No substances	12/20 (60%)	4/7 (57.1%)	7/13 (53.8%)	1^b^

Abbreviations: BD, Bipolar Disorder; BDI, Beck Depression Inventory; BDHI, Buss Durkee Hostility Inventory; HAMA, Hamilton Anxiety Rating Scale; MADRS initial, Montgomery Åsberg Rating Scale at initiation of the clinical trial; YMRS, Young Mania Rating Scale.

Continuous measures are listed as mean and standard deviations and categorical variables listed as number of participants and percentage.

^a^Calculated using 2 tailed *t* test.

^b^Calculated using Fisher’s Exact Test.

### Association **With** Clinical Characteristics

**Figure 1.**
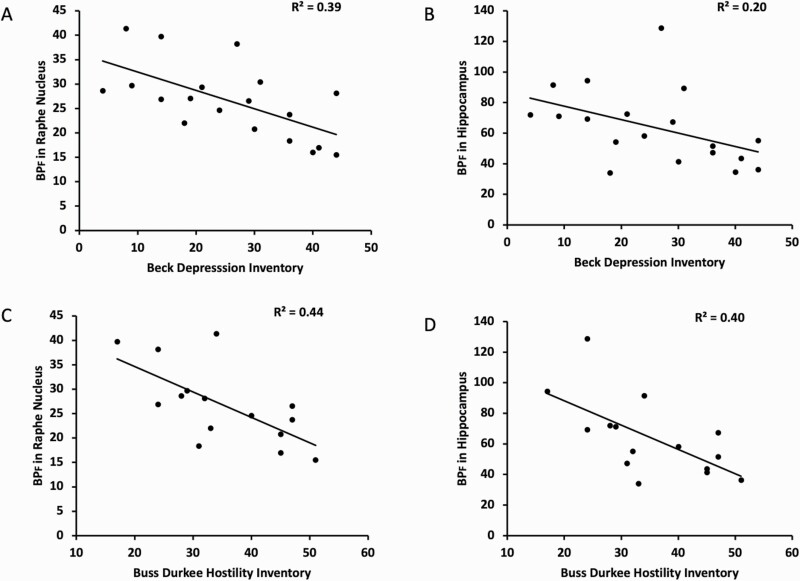
BP_F_ values of [^11^C]CUMI-101 had an inverse association with both a self-reported severity of depression severity as measured by the Beck Depression Inventory (A,B), and the Buss Durkee Hostility Score, a self-reported assessment of lifetime hostility (C,D). (A,C) BP_F_ values within the raphe nucleus region of interest. (B,D) BP_F_ values within a representative region where the serotonin 1A receptors are post synaptic, the hippocampus.

**Figure 2.**
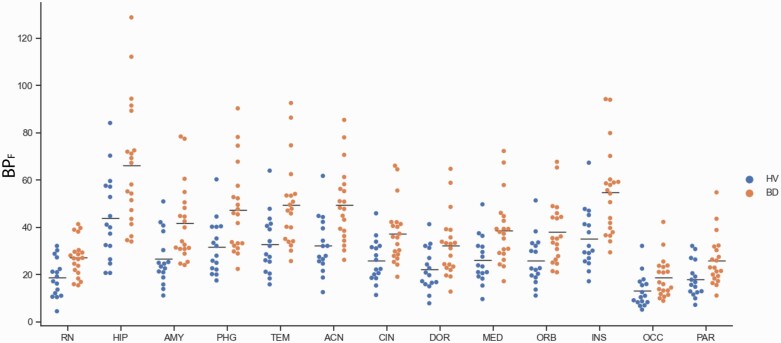
BP_F_ values of [^11^C]CUMI-101 were higher in bipolar disorder (BD) participants (n = 20) compared with healthy volunteers (HVs) (n = 16) in the raphe nucleus (RN) as well as regions where the serotonin 1A receptors are post synaptic. Abbreviations: ACN, anterior cingulate cortex; AMY, amygdala; BD, bipolar disorder participants; CIN, cingulate; DOR, dorsolateral prefronal cortex; HIP, hippocampus; HV, healthy volunteer; INS, insula; MED, medial prefrontal cortex; OCC, occipital lobe; ORB, orbital cortex; PAR, parietal lobe; PHG, parahippocampal gyrus; TEM, temporal lobe. The horizontal bar represents the mean value.

BP_F_ of [^11^C]CUMI-101 within the RN was inversely related to both the pretreatment MADRS score (F = 5.9; df = 1,18; *P* = .026) and BDI score at the time of PET imaging (Fig. 1; F = 12.8; df = 1,17; *P* = .0023). The association with BDI was also observed when all regions in which the 5HT1ARs were post synaptic were included in a linear mixed model (Fig. 1; F = 8.90; df = 1,17; *P* = .0083), although the association with pretreatment MADRS was not (F = 3.8; df = 1,18; *P* = .068). BP_F_ of [^11^C]CUMI-101 within the RN was inversely related to BDHI score (Fig. 2; F = 10.88; df = 1,13; *P* = .0058). This association was also observed when all regions in which the 5HT1ARs are post synaptic were included in a linear mixed model (F = 8.62; df = 1,13; *P* = .0116). BP_F_ of [^11^C]CUMI-101 was not significantly associated with HAM-A scores in the RN only (F = 3.20; df = 1,18; *P* = .091) or when all regions were considered together (F = 2.27; df = 1,18; *P* = .149). All the significant associations remain after Bonferroni correction for multiple comparisons, with the exception of the association with pretreatment MADRS score.

**Figure 3.**
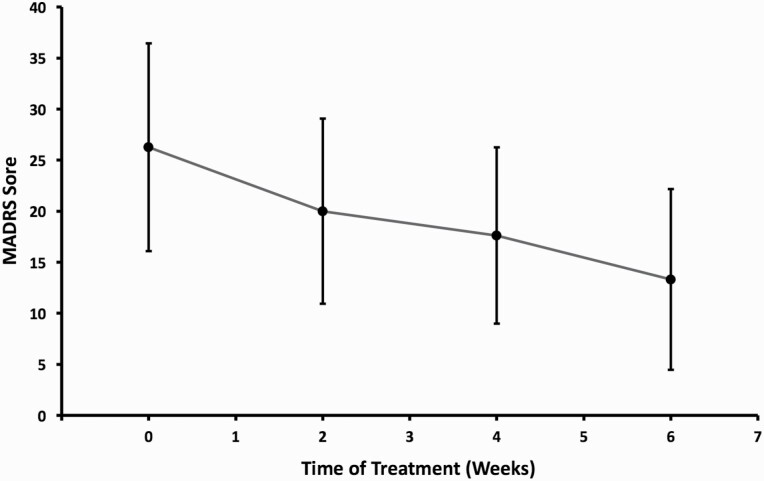
Clinical antidepressant response to 6-week open label SSRI treatment for bipolar depression when added to a mood stabilizer. Abbreviations: MADRS, Montgomery Åsberg Depression Rating Scale. Mean values are plotted and error bars indicate standard deviations.

A secondary analysis found that BP_F_ of [^11^C]CUMI-101 RN autoreceptor binding potential was higher in the BD group (18.7 ± 8.1) compared with HVs (27.2 ± 7.9) (Figure 3; F = 10.43; df = 1,34; *P* = .00275). The same result was found when all regions where the 5HT1ARs are post synaptic were included in a linear mixed model (F = 11.42; df = 1,34; *P* = .00183).

### Clinical Treatment Outcome

For BD participants, week 0 MADRS scores (27 ± 8.8) were significantly higher than MADRS score at week 6 (15.2 ± 10.6, *P* = 4.72e-5). Seven participants (35%) were SSRI responders, defined by >50% decrease in their MADRS score by the end of their treatment course. The average antidepressant response curve across participants is depicted in Figure 3. Fifteen of the participants completed 6 weeks of treatment, and 5 participants completed 4 weeks. The average end SSRI dose was 30 mg/d (±10.8 mg/d).

### Prediction of Clinical Response

**Figure 4.**
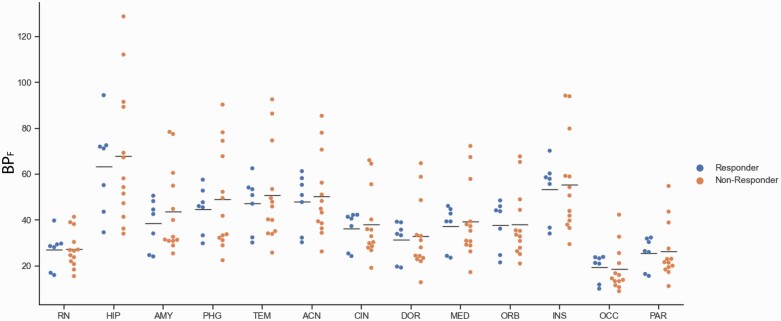
Pretreatment BP_F_ values of [^11^C]CUMI-101 did not differ between responders (n = 7) and non-responders (n = 13) to selective serotonin reuptake inhibitor treatment. (A) BP_F_ values within the raphe nucleus region of interest. (B) BP_F_ values within a representative region where the 5HT1ARs are post synaptic, the hippocampus. Abbreviations: ACN, anterior cingulate cortex; AMY, amygdala; CIN, cingulate; DOR, dorsolateral prefronal cortex; HIP, hippocampus; INS, insula; MED, medial prefrontal cortex; OCC, occipital lobe; ORB, orbital cortex; PAR, parietal lobe; PHG, parahippocampal gyrus; TEM, temporal lobe. The horizontal bar represents the mean value.

Pretreatment BP_F_ of [^11^C]CUMI-101 in the RN did not differ between responders to SSRI treatment and non-responders (Fig. 4; F = 0.014; df = 1,18; *P* = .907). When all regions where the 5HT1ARs are post synaptic were included in a model, again no differences were found between responders and non-responders (F = 0.0019; dF = 1,18; *P* = .966). Pretreatment BP_F_ in the RN was not predictive of end point MADRS score when initial MADRS score was included as a covariate (F = 0.318; df = 1,17; *P* = .580).

### Assessment of Potential Confounds

Neither age nor sex was significant in any of our region of interest statistical models, so the results are reported without these covariates included. Post hoc assessment of binding potential was performed (Table 2). BDHI scores had a positive correlation with f_P_ within the BD group (R = 0.63, *P* = .01), and BDI scores showed a trend toward positive correlation with f_P_(R = 0.42, *P* = .07). BDI scores and BDHI scores had a positive association with each other (R = 0.60, *P* = .02).

**Table 2.** Associations Between Clinical Variables and PET Imaging Parameters

	Injected dose	Injected mass	fp	Reference VT
Responder status	*P* = .14^a^	*P* = .83^a^	*P* = .82^a^	*P* = .86^a^
BDI (n = 17)	R = −0.20, *P* = .44^b^	R = 0.31, *P* = .23^b^	R = 0.42, *P* = .07^b^	R = −0.16, *P* = .54^b^
BDHI (n = 15)	R = −0.11, *P* = .69^b^	R = 0.01, *P* = .97^b^	R = 0.63, *P* = .01^b^	R = 0.06, *P* = .83^b^
HAMA (n = 18)	R = 0.35, *P* = .15^b^	R=−0.14, *P* = .58^b^	R = 0.32, *P* = .17^b^	R = 0.01, *P* = .97^b^

Abbreviations: BDI, Beck Depression Inventory; BDHI, Buss Durkee Hostility Inventory; HAMA, Hamilton Anxiety Rating Scale; fp, Free fraction of radiotracer; Reference VT, VT in the cerebellar grey matter region of interest that was used as the reference region; Responder status, comparison of responders to non-responders.

^a^Calculated using 2 tailed *t* test.

^b^Calculated using Fisher’s Exact Test.

### Whole**-**Brain Voxel-Wise Analyses

**Figure 5.**
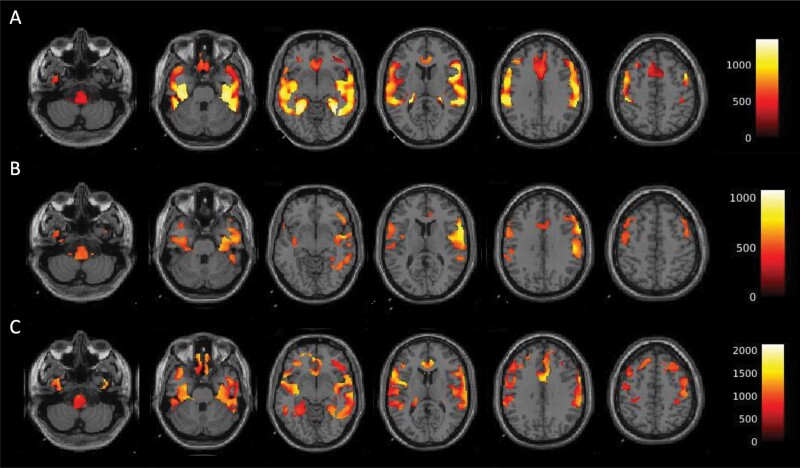
Results from whole-brain, voxel-wise analyses of BP_F_ values of [^11^C]CUMI-101 with clinical variables. (A) BP_F_ of [^11^C]CUMI-101 was higher in bipolar participants (n = 20) compared with healthy volunteers (n = 16). (B) BP_F_ of [^11^C]CUMI-101 was inversely associated with Beck Depression Inventory (n = 19) within the bipolar group. BP_F_ of [^11^C]CUMI-101 was inversely associated with Buss Durkee Hostility Inventory (n = 16). The clusters of significant association between BP_F_ of [^11^C]CUMI-101 and these clinical variables had overlapping regional distribution.

Voxel-wise analyses confirmed the ROI-based results above. BP_F_ of [^11^C]CUMI-101 was higher in BD participants compared with HVs in multiple regions of the temporal cortex, hippocampus, anterior cingulate, dorsolateral prefrontal cortex, and brainstem (Fig. 5A). Within the BD participants, the BP_F_ values were inversely associated with BDI and BDHI scores with a similar distribution, although not as widespread (Fig. 5B–C). No clusters were found to be higher in HVs compared with BD participants, and no positive associations were found between BP_F_ of [^11^C]CUMI-101 and either BDI or BDHI scores. No clusters of BP_F_ of [^11^C]CUMI-101 difference were found between responders and non-responders to treatment and also no associations between BP_F_ of [^11^C]CUMI-101 and HAM-A scores.

## DISCUSSION

These data indicate an association between serotonin signaling, as measured by [^11^C]CUMI-101 5HT1AR PET imaging, and clinical symptomatology of bipolar depression. Specifically, 5HT1AR BP_F_ had an inverse association with depression severity and lifetime history of aggression. A secondary analysis found BP_F_ to be higher in BD participants compared with HVs. [^11^C]CUMI-101 5HT1AR PET imaging did not predict antidepressant treatment response to SSRIs.

The inverse correlation of 5HT1AR binding potential with depression severity is consistent with preclinical studies that found lower 5HT1AR levels in animal models of depression (Shively et al., 2006), including response to stress (Lopez et al., 1999; Richardson-Jones et al., 2010) and emotional deprivation during development (Spinelli et al., 2010). These results differed from previous PET studies of BD that used a different 5HT1AR radiotracer, [^11^C]WAY-100 635, as they reported no associations between binding and depression severity (Sullivan et al., 2009; Nugent et al., 2013b). The discrepancy may be explained by differences between the radiotracers. [^11^C]CUMI-101 is an agonist for the 5HT1AR in cell culture, in contrast to the antagonist radiotracer [^11^C]WAY-100 635 (Hendry et al., 2011). The agonist property of [^11^C]CUMI-101 was not replicated in brain homogenate studies (Shrestha et al., 2011), consistent with [^11^C]CUMI-101 as a biased agonist. Assuming [^11^C]CUMI-101 does have agonist properties in vivo, depressive symptom severity in BD may be related to lower agonist affinity of [^11^C]CUMI-101, an association that would not be detectable by the antagonist ligand [^11^C]WAY-100 635.

Our finding of an inverse correlation between BP_F_ of [^11^C]CUMI-101 and lifetime aggression severity score was consistent with a previous study in HVs (Parsey et al., 2002). Consistent with our finding, 5HT1AR agonist medications reduce aggressive behavior both in animal models and HVs, indicating that lower 5HT1AR neurotransmission is associated with aggression (Olivier et al., 1995; de Boer and Koolhaas, 2005). Conversely, depleting serotonin in animal models can increase aggressive behavior (Valzelli et al., 1981). Those preclinical data suggest that the lower [^11^C]CUMI-101 BP_F_ reported here may indicate lower serotonin signaling in those participants with more aggression. Aggressive behavior has clinical importance in BD, as it has been associated with a poor clinical course, including risk of alcohol use disorders and suicide attempts (Khalsa et al., 2018). We did find a positive association between the BDHI score and the BDI scale, and we cannot rule out the possibility that the participants’ depression symptoms could have impacted their responses to the BDHI questions.

We did not detect a significant association between BP_F_ of [^11^C]CUMI-101 and symptoms of anxiety within BD participants, although a trend association was found with data from the RN, as defined as *P* < .1. The observation should be followed-up in a larger study with greater statistical power. Studies of 5HT1AR knockout mice consistently report greater symptoms of anxiety compared with wild-type mice (Heisler et al., 1998; Parks et al., 1998; Ramboz et al., 1998; Klemenhagen et al., 2006). Similarly, past studies of anxiety disorders have reported lower [^11^C]WAY-100 635 binding when compared with HVs (Lanzenberger et al., 2007; Nash et al., 2008). [^11^C]WAY-100 635 binding was also associated with anxiety in HVs and major depressive disorder participants (Tauscher et al., 2001; Sullivan et al., 2005).

Our study was limited by the mild to moderate depression severity of participants, and studies with a greater range of symptom severities are warranted. The BD participants in the current study were taking a mood stabilizer medication at the time of scanning. One previous study found that treatment with either divalproex or lithium in BD caused an increase in [^11^C]WAY-100 635 binding (Nugent et al., 2013a). It is possible that the mood stabilizer treatment before scanning effected both baseline clinical ratings and 5HT1AR binding. Alternately, it is possible that variance in the mood stabilizer’s effects on 5HT1AR binding introduced noise into the analysis of baseline data. However, neither of these scenarios would confound our interpretation of the results that there is an association between the symptoms and baseline 5HT1AR binding.

We previously reported higher BP_F_ of [^11^C]WAY-100 635 binding in the brain of depressed BD participants compared with HVs (Sullivan et al., 2009). Our results here are consistent with those data. However, another study found no differences between depressed BD and HV groups using [^11^C]CUMI-101 and BP_F_ (Ananth et al., 2020). Our diagnostic group results were obtained through a secondary analysis, and the HV participants were recruited as part of other studies. The analysis could not control for an effect of the mood stabilizer medications on BP_F_ of [^11^C]CUMI-101 in the bipolar participants, so we cannot rule out the possibility that the diagnostic differences are driven by the mood stabilizer treatment.

PET signal can be affected by either the density of receptors that are available to interact with a radiotracer or the affinity of the receptors to bind to a radiotracer. Our data could therefore be explained by a model in which there is an upregulation of the 5HT1ARs that occurs in BD compared with HVs but that a conformational change of the 5HT1ARs occurs when depression symptoms arise, which causes less affinity to the [^11^C]CUMI-101 radiotracer. In this model, the association between PET signal and depression severity was not seen using the [^11^C]WAY-100 635 radiotracer because the 2 radiotracers have different binding characteristics to the 5HT1AR. An alternative model to explain our data would be that the upregulation of 5HT1ARs is a compensatory effect in BD that decreases as depressive symptoms occur.

We did not find that [^11^C]CUMI-101 5HT1AR PET imaging predicted antidepressant treatment response to SSRIs. The latter result differed from our previous study in BD depression, where higher pre-treatment BP_F_ of [^11^C]WAY-100 635 predicted remission to unrestricted treatment (Lan et al., 2013). It also differed from a recent finding that lower pre-treatment BP_F_ of [^11^C]CUMI-101 predicted a better antidepressant response to lithium monotherapy (Ananth et al., 2020). These data raise the possibility that although the baseline level of 5HT1AR binding does not predict clinical improvement in general, it may predict the response to certain medications. Lithium has been found to increase serotonin release (Treiser et al., 1981; Scheuch et al., 2010), a process that may be predicted by 5HT1AR binding better than the mechanism of action of SSRIs, the inhibition of serotonin reuptake. An analogous study to this one in major depressive disorder, however, reported that higher BP_F_ of [^11^C]WAY-100 635 in the RN was associated with remission to an SSRI medication, a result that was not replicated in BD here (Miller et al., 2013). As the bipolar participants were on mood stabilizer medications during the study, we cannot rule out the possibility that differences in the mood stabilizer treatment affected the clinical response to the SSRI. Previous clinical trials of fluoxetine in BD depression reported higher rates of response than we found here (Cohn et al., 1989; Simpson and DePaulo, 1991; Amsterdam and Shults, 2010), although at least 1 trial reported a comparable response rate with our study of 38% (Amsterdam et al., 2004). Our results may therefore imply that 5HT1AR PET did not predict clinical outcome because our sample was particularly antidepressant treatment resistant.

BP_F_ was our a priori outcome measure. Incorporating serum activity and tracer’s free fraction (f_P_) were particularly important for the study because the BD participants were on medications at the time of PET scanning that could have affected either the metabolism of the radiotracer or its protein binding. We found that the f_P_ values of [^11^C]CUMI-101 had a positive association with aggression severity and a trend toward a positive association with depression severity in the BD participants (Table 2). We cannot rule out the possibility that depression severity had an impact on radiotracer’s f_P_, indicating the importance of considering f_P_ when quantifying [^11^C]CUMI-101 in this population.

Our results indicate the importance of serotonin signaling to the pathophysiology of depression in BD and imply that serotoninergic pathways may form a therapeutic target for future medications. Of note, the second-generation antipsychotics that are approved for the treatment of depression in BD have serotonin 1A receptor agonist properties, and future studies could determine the importance of that agonism to their antidepressant effect. Additional studies could also discern whether a disruption to serotonin signaling occurs during mania or hypomania. It would also be important to delineate how a disruption in serotonin signaling occurs in relation to other neuropathological processes. Our data contribute to the existing body of knowledge that BD involves molecular and cellular changes in the brain of individuals afflicted by the disorder.

## Interest Statement

Dr Mann receives royalties from the Research Foundation for Mental Hygiene for commercial use of the C-SSRS. No other authors report conflicts of interest.
